# Engineered Nature‐Based Sonosensitizer for Broad‐Spectrum Infectious Tissue Repair via Sono‐Ionic Therapy

**DOI:** 10.1002/exp2.70208

**Published:** 2026-08-10

**Authors:** Liang Ma, Hongchuan Wang, Weidong Liang, Tao Xu, Jie Lei, Li Zhang, Shutao Gao, Xiaoyu Cai, Qian Li, Wenzhao Wang, Chuanhui Xun, Weibin Sheng, Cao Yang, Yingsong Wang

**Affiliations:** ^1^ Department of Orthopaedics Union Hospital Tongji Medical College Huazhong University of Science and Technology Wuhan China; ^2^ Department of Spine Surgery The First Affiliated Hospital of Xinjiang Medical University Urumqi Xinjiang China; ^3^ Department of Orthopedics The Second Affiliated Hospital of Kunming Medical University Kunming Yunnan China; ^4^ Department of Chemical & Biomolecular Engineering University of Notre Dame Indiana USA; ^5^ School of Biomedical Sciences The Chinese University of Hong Kong Hong Kong SAR China

**Keywords:** bacterial elimination, infectious tissue repair, sono‐ionic therapy, sonosensitizers, stem cell

## Abstract

Repairing infectious tissues, particularly bone and wound infections, poses significant challenges to clinicians due to the lack of effective treatment modalities. Sonodynamic therapy, which utilizes sonosensitizers to generate reactive oxygen species (ROS) under ultrasound (US) energy for antibacterial purposes, still lacks suitable sonosensitizers with good biocompatibility that can simultaneously promote the repair of various infected tissues. In this study, we constructed a novel sonosensitizer composed of hemoglobin derived from nature and zeolitic imidazolate framework (ZIF)‐8, which exhibits excellent biocompatibility and activity. This sonosensitizer enables early‐stage high‐power US to generate substantial ROS for antibacterial effects and late‐stage low‐power sono‐ionic therapy for tissue repair. We employed bone, wound infection, and wound biofilm models to evaluate the therapeutic efficacy in different infected tissues. We utilized microneedles to assemble nanoparticles to match the different disease models, achieving deep biofilm clearance. Furthermore, to investigate the repair mechanisms, we developed a bioadhesive‐derived hydrogel loaded with nanoparticles for wound treatment. RNA sequencing revealed that sono‐ionic therapy promotes tissue repair through the peroxisome proliferator‐activated receptor signaling pathway. Therefore, our platform holds great potential for broad‐spectrum treatment of infectious tissue repair.

## Graphical abstract

A schematic illustration of an engineered nature‐based sonosensitizer enables early‐stage high‐power ultrasound to generate substantial reactive oxygen species for antibacterial effects and late‐stage low‐power sono‐ionic therapy for infected bone repair, infected wound biofilm therapy, and wound regeneration.

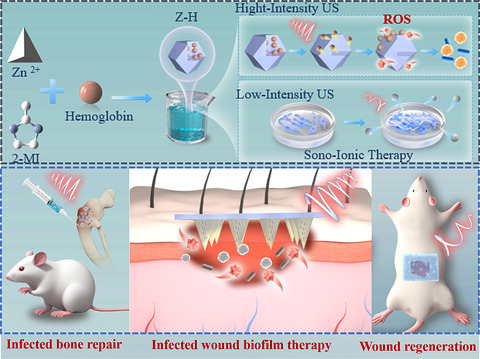

**Abbreviations**
^1^O_2_
: singlet oxygen
ALP: alkaline phosphatase
ARS: alizarin red S
BV/TV: bone volume fraction
CCK‐8: Cell Counting Kit‐8
CLSM: confocal laser scanning microscope
COL1: Collagen I
DEGs: differentially expressed genes
DI: deionized
DPBF: 1,3‐diphenylisobenzofuran
EIS: electrochemical impedance spectroscopy
FE‐SEM: field‐emission scanning electron microscopy
GO: gene ontology
HA MNs: hyaluronic acid microneedles
Hb: hemoglobin
hBMSCs: human bone marrow mesenchymal stem cells
HmIM: 2‐methylimidazole
HRTEM: high‐resolution transmission electron microscopy
IF: immunofluorescence
IL: interleukin
INOS: inducible nitric oxide synthase
KEGG: Kyoto Encyclopedia of Genes and Genomes
KGM‐XG: konjac glucomannan‐xanthan gum
LB: Luria–Bertani
MF: monoformazan
MRSA: methicillin‐resistant *Staphylococcus aureus*
NBT: nitroblue tetrazolium chloride
•O_2_‐: superoxide anion
OCN: osteocalcin
OPN: osteopontin
PCA: principal component analysis
PPAR: peroxisome proliferator‐activated receptor
qRT‐PCR: quantitative reverse transcription polymerase chain reaction
ROS: reactive oxygen species
RUNX2: Runt‐related transcription factor 2
SDT: Sonodynamic therapy
TNF‐*α*
: tumor necrosis factor *α*
US: ultrasound
Z‐H NPs: ZIF‐8‐hemoglobin nanoparticles
Z‐H@HA MNs: Z‐H‐loaded HA microneedles
Z‐H@KGM‐XG: Z‐H‐loaded KGM‐XG hydrogel
ZIF‐8: zeolitic imidazolate framework‐8
*α*‐SMA: *α*‐smooth muscle actin

## 1 Introduction

Infectious diseases caused by multidrug‐resistant bacteria, such as methicillin‐resistant *Staphylococcus aureus* (MRSA), pose a significant global public health challenge [1, 2, 3]. These infections not only cause immense patient suffering but also impose a heavy socioeconomic burden by prolonging hospitalization and increasing the risk of resistant bacteria spread [4, 5]. Traditional treatments, including antibiotic therapy and surgical debridement, face multiple challenges. Overuse of antibiotics accelerates bacterial resistance and bioaccumulation [6, 7], while the formation of bacterial biofilms in chronic infections hinders drug penetration and fails to achieve simultaneous infection control and tissue regeneration [8, 9, 10]. Although debridement removes infected and necrotic tissues, it fails to promote functional regeneration and often damages surrounding healthy tissues and the local microenvironment, further delaying healing [11, 12, 13]. These shortcomings highlight the urgent need for novel therapeutic strategies to address complex infections and tissue regeneration [14, 15].

In recent years, sonodynamic therapy (SDT) has emerged as a promising non‐invasive approach due to its deep tissue penetration and dual functionality in antimicrobial activity and tissue repair promotion [16, 17]. SDT employs ultrasound (US) to activate sonosensitizers, generating reactive oxygen species (ROS) that kill pathogens through oxidative damage to bacterial membranes, proteins, and DNA, while avoiding resistance development [18, 19, 20, 21]. Additionally, US exhibits excellent tissue penetration capabilities, enabling it to act efficiently on deep tissues. Its highly focused properties ensure minimal collateral damage to surrounding healthy tissues [22, 23]. Notably, using low‐intensity US, the sonosensitizers generate microcurrents, which synergistically enhance antibacterial effects and stimulate tissue repair pathways, such as epidermal regeneration, osteoblast differentiation, and angiogenesis. This dual mechanism addresses the limitations of conventional treatments, offering a comprehensive strategy for infectious disease management [24, 25, 26, 27, 28]. The efficacy of SDT critically depends on the performance of sonosensitizers. Commonly used sonosensitizers are categorized into organic (e.g., porphyrin derivatives) and inorganic (e.g., TiO_2_ and ZnO nanoparticles) [29, 30]. Organic sonosensitizers, such as porphyrin compounds, exhibit strong sonodynamic activity but suffer from poor water solubility, rapid metabolism, poor biochemical stability, and high phototoxicity [31, 32]. Moreover, their tendency to aggregate through *π*–*π* stacking, a non‐covalent interaction between aromatic rings, reduces the efficient generation of ROS. This aggregation limits the surface area available for interaction with US and reduces the overall effectiveness of organic sonosensitizers in SDT [33, 34, 35]. Despite the high stability of inorganic sonosensitizers, they exhibit limitations, including suboptimal biocompatibility and challenges associated with in vivo degradation [36, 37, 38]. Thus, developing novel sonosensitizers with high ROS generation, controlled release, and biosafety is pivotal for SDT clinical translation.

Hemoglobin (Hb), a natural metalloporphyrin protein, offers unique advantages as a sonosensitizer [39]. It consists of four polypeptide chains and four heme groups (iron‐based porphyrins), with each polypeptide chain bound to a heme group to form a monomer or subunit [40, 41, 42]. This structural arrangement enables the separation of the four porphyrin rings by the polypeptide chains, thereby preventing the aggregation of porphyrin molecules, which could otherwise lead to reduced ROS production [43]. Moreover, Hb plays a crucial role in alleviating the hypoxic microenvironment, which is often a significant challenge in the treatment of infected tissues. Hb's inherent capacity as a natural oxygen carrier enables the controlled release of oxygen, which is essential for enhancing the generation of ROS under SDT [44, 45, 46, 47]. This oxygenation effect not only potentiates antimicrobial activity but also accelerates tissue regeneration by modulating the local microenvironment [48, 49, 50, 51]. Additionally, Hb exhibits excellent biocompatibility, superior water solubility, and low phototoxicity, rendering it an ideal candidate for sonosensitizers [52]. However, free Hb is susceptible to proteolytic degradation in physiological conditions. Therefore, it is imperative to develop stable encapsulation and targeted delivery systems using nanocarriers. Zeolitic imidazolate frameworks (ZIFs) are a class of metal‐organic frameworks composed of metal ions coordinated with imidazolate linkers. These materials possess a highly ordered structure with large surface areas, tunable pore sizes, and excellent stability under physiological conditions. ZIF‐8, a representative member of this class, serves as an excellent drug delivery carrier due to its unique physicochemical properties [53, 54]. ZIF‐8 is synthesized through the coordination of zinc ions and 2‐methylimidazole (HmIM) [55], exhibiting excellent biocompatibility and pH‐responsive degradation characteristics [56, 57]. Under neutral physiological conditions, it maintains a stable structure; however, in the acidic microenvironment at infection sites, ZIF‐8 gradually degrades, enabling precise release of its encapsulated drugs [58]. Additionally, during the degradation process, ZIF‐8 releases a significant amount of zinc ions [59], which not only exhibit antibacterial activity but also promote cell adhesion, migration, proliferation, and differentiation by activating multiple signaling pathways [60, 61, 62]. This accelerates the healing of bone defects and skin wounds while mitigating excessive inflammatory responses [63, 64, 65, 66]. Consequently, the encapsulation of Hb within ZIF‐8 not only significantly enhances its stability in vivo, thereby improving its sonocatalytic activity [67], but also allows the released zinc ions to synergize with the microcurrent generated by SDT, further facilitating the repair of bone, skin, and other tissue injuries through sono‐ionic therapy.

In this study, we developed Z‐H nanoparticles (Z‐H NPs) by encapsulating Hb within ZIF‐8. In order to further verify its sono‐ionic therapy ability, we developed three infectious animal models of bone, wound infection, and wound biofilm and loaded Z‐H into hyaluronic acid microneedles (HA MNs) for wound biofilm therapy and konjac glucomannan‐xanthan gum (KGM‐XG) hydrogels for treating skin wounds. With its minimally invasive penetration capability, HA MNs can effectively penetrate the bacterial biofilm barrier associated with chronic wound infections, thereby facilitating targeted delivery of Z‐H NPs [68, 69]. The rapid dissolution characteristics of HA MNs ensure sustained release of Z‐H NPs at the wound site while preventing tissue damage that may result from traditional injection methods [70]. Additionally, KGM‐XG hydrogels, characterized by their bionic porous structure and superior water retention properties, create a moist microenvironment conducive to skin wound repair [71, 72]. This promotes cell migration and angiogenesis and enhances the localized retention of Z‐H NPs [73, 74].

**SCHEME 1:**
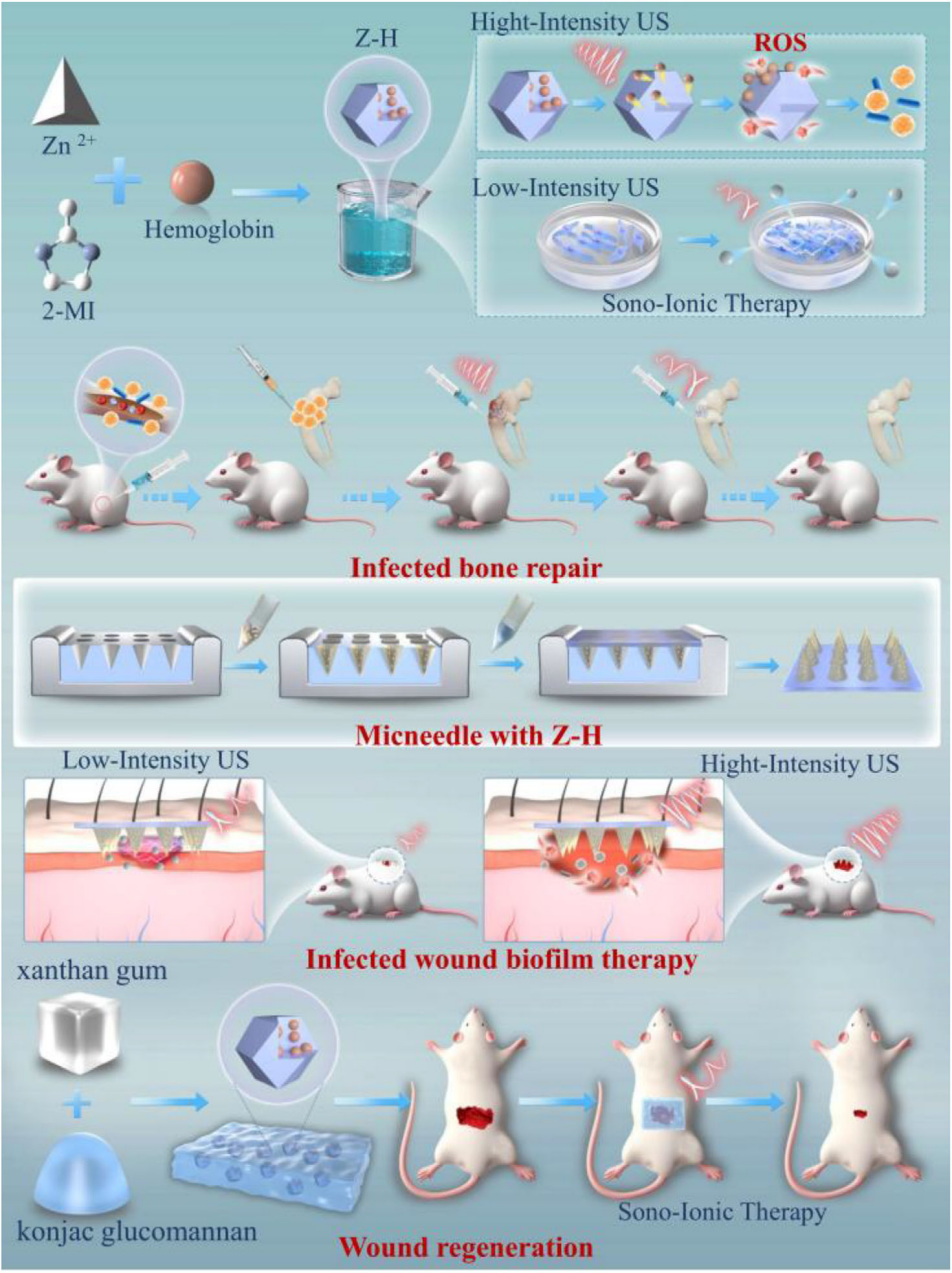
Schematic illustration of nature‐based sonosensitizer for broad‐spectrum infectious tissue repair via sono‐ionic therapy.

Experimental results show that Z‐H NPs, under high‐intensity US, efficiently generate high levels of ROS (^1^O_2_ and •O_2_‐) for rapid bacterial eradication. Simultaneously, low‐intensity US‐triggered microcurrent stimulation and sustained zinc ion release activate tissue repair‐related signaling pathways (e.g., peroxisome proliferator‐activated receptor [PPAR] signaling), modulate the inflammatory microenvironment, and promote tissue regeneration at infection sites [75]. This “bactericidal‐anti‐inflammatory‐regenerative” multi‐mechanistic approach enables the effective treatment of tissue infections. In infected bone defect and skin wound models, Z‐H/US combination therapy significantly increased bone volume fraction (BV/TV), accelerated wound healing substantially, and markedly reduced levels of inflammatory factors such as TNF‐*α*. In the bacterial biofilm infection model of skin wounds, Z‐H‐loaded HA MNs (Z‐H@HA MNs) successfully eradicated MRSA biofilms in chronic skin infection wounds under ultrasonic intervention. Similarly, combining Z‐H@KGM‐XG hydrogel and US therapy enhanced collagen deposition rates and angiogenesis in a skin wound model. Transcriptomic analysis suggested that Z‐H@KGM‐XG Hydrogel under US might promote wound repair by regulating cellular responses to external stimuli and activating the PPAR pathway, a key regulator of cellular metabolism and inflammation. PPARs are a group of nuclear receptors that play a critical role in the regulation of various biological processes, including lipid metabolism, immune response, and tissue regeneration. Activation of PPAR signaling has been shown to promote tissue repair by modulating inflammatory responses, enhancing cell differentiation, and stimulating the formation of new blood vessels, all of which are crucial for efficient wound healing [75, 76, 77, 78]. This study provides a novel strategy for developing multifunctional nanomaterials that integrate antimicrobial and regenerative functions, thereby advancing the clinical translation of SDT for the treatment of infectious diseases (See Scheme 1).

## 2 Materials and Methods

### 2.1 Materials

Zinc acetate (99%, C_4_H_6_O_4_Zn·2H_2_O), Hb, XG, and KGM were purchased from Aladdin. HmIM was purchased from Sigma‐Aldrich. Sodium hyaluronate (HA) was purchased from Shaanxi Baichuan Biotechnology Co., Ltd.

### 2.2 Synthesis of ZIF‐8 Nanoparticles

The preparation of ZIF‐8 nanoparticles was initiated by dispersing 44 mg of zinc acetate in 1 mL of deionized (DI) water. Subsequently, a 3 mL solution of HmIM at a concentration of 160 mg mL^−1^ was prepared. The two solutions were then mixed and stirred at room temperature for 6 h to facilitate the formation of ZIF‐8 nanoparticles. The nanoparticles were subsequently harvested by centrifugation at 10,000 rpm for 10 min. Afterward, the nanoparticles were washed with ethanol three times to remove any residual reactants and then dried in vacuum at 80°C for 12 h to obtain the final product.

### 2.3 Preparation of Z‐H Nanoparticles

For the synthesis of ZIF‐8‐hemoglobin (Z‐H) nanoparticles, 44 mg of zinc acetate was initially dispersed into 1 mL of DI water containing varying amounts of Hb (1 mg, 2 mg, 4 mg, and 6 mg). Subsequently, 3 mL of an HmIM solution (160 mg mL^−1^) was added. The resulting mixture was stirred at room temperature for 6 h to allow for the formation of Z‐H nanoparticles. The nanoparticles were then collected by centrifugation at 8000 rpm for 10 min. Afterward, the precipitate was washed three times with DI water. The nanoparticles were subsequently freeze‐dried to obtain Z‐1H, Z‐2H, Z‐4H, and Z‐6H nanoparticles, with each sample corresponding to the different initial Hb concentrations.

### 2.4 Preparation of HA Microneedles (HA MNs) and Z‐H‐Loaded HA Microneedles (Z‐H@HA MNs)

The fabrication of HA‐MNs and Z‐H@HA‐MNs was performed using a solution‐casting method. Initially, 2 g of HA powder was dissolved in 10 mL of DI water (for HA‐MNs preparation) or 10 mL of Z‐6H‐containing DI water (500 µg mL^−1^, for Z‐H@HA‐MNs preparation) under continuous magnetic stirring at room temperature for 1 h to obtain homogeneous and stable solutions. The prepared solutions were then carefully dispensed onto polydimethylsiloxane microneedle molds. The molds were subsequently placed in a vacuum drying oven and subjected to vacuum treatment for 5 min. This vacuum‐assisted filling process was repeated three times to ensure complete filling of the microneedle cavities and effective removal of air bubbles. Finally, the molds were transferred to a drying oven and maintained at a temperature below 40°C for overnight curing to facilitate solidification of the microneedles. After drying, the solidified microneedles were meticulously detached from the molds using precision tweezers.

### 2.5 Preparation of KGM‐XG Hydrogel and Z‐H@KGM‐XG Hydrogel

The fabrication process of KGM‐XG hydrogel and Z‐H‐loaded KGM‐XG hydrogel (Z‐H@KGM‐XG Hydrogel) is outlined as follows. Initially, Z‐6H was dispersed in DI water to prepare a Z‐6H solution with a concentration of 500 µg mL^−1^. Subsequently, XG and KGM were separately dissolved in either DI water (for KGM‐XG hydrogel preparation) or the prepared Z‐6H solution (for Z‐H@KGM‐XG hydrogel preparation) under continuous magnetic stirring, yielding 10 mg mL^−1^ solutions of both polymers. The prepared XG and KGM solutions were then mixed at a volume ratio of 2:3, followed by heating at 80°C with constant stirring for 30 min to ensure complete homogenization. Finally, the mixed solution was transferred into a 48‐well plate and allowed to undergo spontaneous gelation at room temperature.

### 2.6 Characterization of Materials

The morphological features of ZIF‐8 and Z‐H nanoparticles were characterized using high‐resolution transmission electron microscopy (HRTEM; JEM‐2100F, JEOL, Japan). The surface morphology of HA MNs and Z‐H@HA MNs, along with freeze‐dried KGM‐XG hydrogel and Z‐H@KGM‐XG hydrogel, was examined using field‐emission scanning electron microscopy (FE‐SEM).

### 2.7 Quantification of Hb Loading in Z‐H

To quantify the drug loading content (LC) of Hb in Z‐H nanoparticles, Hb was first completely dissolved in 0.1 M HCl and then diluted to a series of concentrations to establish a calibration curve by measuring the UV–Vis absorbance at 400 nm. Subsequently, Z‐H nanoparticles were also completely dissolved in 0.1 M HCl. After centrifugation at 15,000 g for 10 min, the supernatants of each solution were collected, and their absorbance at 400 nm was recorded. The Hb content in the nanoparticles was calculated from the calibration curve. LC was determined using the following equation: LC (%) = (weight of Hb in Z‐H/total weight of Z‐H) × 100%. The encapsulation efficiency (EE) was determined as follows: EE (%) = (weight of Hb in Z‐H/total weight of Hb initially added) × 100%.

### 2.8 In Vitro Antibacterial Assay

The antibacterial efficacy of Z‐H nanoparticles (500 µg mL^−1^) was evaluated using the spreading plate method. Briefly, MRSA suspensions (10^9^ CFU mL^−1^) were diluted 200‐fold using either saline or Z‐H‐containing saline solutions (500 µg mL^−1^). Subsequently, 200 µL of each diluted suspension was subjected to US (1.0 MHz, 1.5 W cm^−2^, 50% duty cycle) irradiation for 15 min using an ultrasonic therapy apparatus. After US treatment, the bacterial suspensions were further diluted 100‐fold with saline, and 20 µL of the diluted suspensions were spread onto standard Luria–Bertani (LB) agar plates. The plates were then incubated at 37°C for 24 h. Finally, the plates were photographed, and the number of colonies was counted to calculate the antibacterial rate for each group.

### 2.9 Bacterial Live/Dead Staining Assay

The viability/cytotoxicity assay kit for bacterial cells (UElandy, China) was employed to conduct the bacterial live/dead staining experiment. Briefly, MRSA suspensions (10^9^ CFU mL^−1^) were diluted 200‐fold using either saline or Z‐H‐containing saline solutions (500 µg mL^−1^). Subsequently, 200 µL of each diluted suspension was subjected to US irradiation for 15 min (1.0 MHz, 1.5 W cm^−2^, 50% duty cycle). Following US treatment, a 100× concentrated dye solution was prepared according to the manufacturer's instructions. The treated bacterial suspensions (200 µL) were mixed with 2 µL of the 100× dye solution and incubated at room temperature in the dark for 15 min. After incubation, the stained samples were washed 2–3 times with saline and resuspended in an appropriate volume of saline. Finally, 5 µL of the stained bacterial suspensions were placed on glass slides, covered with coverslips, and immediately observed under a confocal laser scanning microscope (CLSM; Olympus, Japan) for image acquisition.

### 2.10 Electrochemical Characterization

The electrochemical properties of ZIF‐8 and Z‐H nanoparticles were assessed in a Na_2_SO_4_ solution (0.1 M) using an electrochemical analyzer (CHI660E, China). The measurements involved the determination of both ultrasonic‐response current and electrochemical impedance spectroscopy (EIS). For sample preparation, 200 µL of ethanol solution containing the nanoparticles (2 mg mL^−1^) was drop‐cast onto indium tin oxide‐coated glass slides and allowed to dry at room temperature for 2 h. Electrochemical measurements were performed using a standard three‐electrode system, with the prepared samples as working electrodes, a platinum wire as a counter electrode, and Ag/AgCl as a reference electrode. The US‐responsive current was recorded under intermittent US stimulation (1.5 W cm^−2^, continuous).

### 2.11 Detection of Reactive Oxygen Species (ROS) Generation

The generation of ROS under US irradiation was investigated using specific chemical probes. For singlet oxygen (^1^O_2_) detection, an ethanol solution of 1,3‐diphenylisobenzofuran (DPBF, 50 µg mL^−1^) was employed. Briefly, 200 µL of aqueous sample solution (1 mg mL^−1^) was mixed with 200 µL DPBF solution. The mixture was subjected to US irradiation (1.0 MHz, 1.5 W cm^−2^, 50% duty cycle). The ^1^O_2_ generation was monitored by tracking the decrease in DPBF absorption peak at 410 nm at 0, 3, 6, 9, and 12 min. For superoxide anion (•O_2_^−^) detection, a dimethyl sulfoxide solution of nitroblue tetrazolium chloride (NBT, 2 µg mL^−1^) was utilized. The •O_2_^−^ radicals reacted with NBT to form monoformazan (MF), which was quantified by measuring the characteristic absorption band of MF.

### 2.12 In Vitro Cytotoxicity Assay

The in vitro cytotoxicity of the materials was evaluated using the Cell Counting Kit‐8 (CCK‐8; HYCEZMBIO, China). Human bone marrow mesenchymal stem cells (hBMSCs) were seeded at a density of 1.5 × 10^4^ cells per well in 24‐well plates and co‐cultured with complete growth medium containing varying concentrations of Z‐6H (0, 5, 10, 20, 30, and 40 µg mL^−1^), with each group featuring three replicates. The cells were cultured for 1, 4, and 7 days before the CCK‐8 assay was performed. The assay procedure was as follows: First, a cell culture medium containing 10% CCK‐8 reagent was prepared. The medium containing the materials was then removed, and the cells were washed three times with sterile PBS. Next, 400 µL of the medium containing 10% CCK‐8 was added to each well and incubated under standard culture conditions in the dark for 2.5 h. The liquid from the 24‐well plates was subsequently transferred to a 96‐well plate, with 100 µL per well. Finally, the absorbance of each well was measured using a microplate reader at 450 nm.

### 2.13 In Vitro Cytocompatibility Assay of Hydrogels

The cytocompatibility of KGM‐XG Hydrogel and Z‐H@KGM‐XG Hydrogel was assessed using the Calcein/PI cell viability/cytotoxicity assay kit (Beyotime, China). After freeze‐drying, the hydrogels were placed into 48‐well plates. hBMSCs were seeded onto the hydrogels at a density of 1 × 10^4^ cells per well. The cells were cultured for 1, 3, and 6 days before performing the live/dead cell staining. Briefly, after aspirating the culture medium, the cells were washed once with PBS. An appropriate volume of the Calcein AM/PI staining solution was then added, and the cells were incubated at 37°C in the dark for 30 min. Following the incubation, the hydrogels were removed and placed onto glass slides. The stained cells were observed and imaged using a CLSM (Olympus, Japan).

### 2.14 Alkaline Phosphatase (ALP) Staining

The alkaline phosphatase (ALP) staining assay was performed across four groups: Control, US, Z‐H, and Z‐H/US. hBMSCs were plated at a density of 1.5 × 10^4^ cells per well in 24‐well plates and co‐cultured with Z‐6H (10 µg mL^−1^) for 21 days prior to ALP staining. During this period, the US and Z‐H/US groups received US treatment (1.0 MHz, 0.2 W cm^−2^, 50% duty cycle) for 10 min every 3 days, totaling four treatments. The procedure for ALP staining is outlined as follows: Cells were rinsed three times with PBS and then fixed with 4% paraformaldehyde for 30 min. Following this, the ALP staining solution (Beyotime, China) was prepared and added to the cells for the staining process. After incubating at room temperature for 30 min, the staining solution was removed, and ultrapure water was added to terminate the staining reaction. Finally, the stained samples were observed under a microscope and photographed to document the results.

### 2.15 Alizarin Red S (ARS) Staining

The experimental groups and cell culture conditions for Alizarin Red S (ARS) staining were identical to those described for ALP staining, including the US treatment protocol. After 21 days of culture, hBMSCs were washed three times with PBS and then fixed with 4% paraformaldehyde for 30 min. Subsequently, the ARS staining solution (Solarbio, China) was added to the cells for staining. After incubating at room temperature for 20 min, the staining solution was aspirated, and the cells were gently washed three times with ultrapure water. Finally, the staining results were observed under a microscope and photographed.

### 2.16 Immunofluorescence (IF) Staining

The immunofluorescence (IF) staining experiment was conducted across four groups: Control, US, Z‐H, and Z‐H/US. hBMSCs were seeded onto cell culture slides in 24‐well plates at a density of 1.5 × 10^4^ cells per well and incubated under corresponding conditions for 21 days. The US and Z‐H/US groups received US treatment every three days (1.0 MHz, 0.2 W cm^−2^, 50% duty cycle) for 10 min per session, totaling four treatments. The IF staining procedure is detailed as follows: Cells were washed three times with PBS and fixed with 4% paraformaldehyde for 30 min. The cells were then permeabilized with 0.5% Triton X‐100 for 15 min. After blocking with immunostaining blocking solution for 30 min, diluted primary antibodies against ALP (Affinity, rabbit source) and OPN (Proteintech, rabbit source) were added to the cell slides and incubated overnight at 4°C in a humidified chamber. After removing the primary antibodies, the cell culture slides were incubated with an adequate amount of diluted anti‐rabbit antibody with a red fluorophore group (Proteintech) in a humidified chamber away from light for 1 h. Following the removal of the secondary antibodies, the FITC‐conjugated phalloidin solution (Solarbio, China) was added for 15 min to stain the cytoskeleton under light protection at room temperature. After discarding the phalloidin solution, a drop of DAPI solution containing an anti‐fluorescence quencher (Beyotime, China) was added to the slides and incubated for 10 min away from light at room temperature to stain the cell nuclei. Finally, the slides were observed and imaged under a CLSM (Olympus, Japan) under light protection conditions.

### 2.17 Quantitative Reverse Transcription Polymerase Chain Reaction (qRT‐PCR)

The qRT‐PCR experiment was conducted across four groups: Control, US, Z‐H, and Z‐H/US. hBMSCs were plated at a density of 6 × 10^4^ cells per well in 6‐well plates and cultured under varying conditions for 14 days. The US and Z‐H/US groups received US treatment every three days (1.0 MHz, 0.2 W cm^−2^, 50% duty cycle) for a total of three treatments, each lasting 10 min. Total RNA was extracted from the cells of each group for subsequent analysis. The specific steps are as follows: After washing the cells three times with PBS, total RNA was extracted from each group using the RNA‐easy kit (Vazyme, R701‐01), followed by isopropanol purification and two washes with 75% ethanol. The RNA was then dissolved in 20–50 µL of DEPC water (Solarbio, China). Then, a 20 µL mixture of RNA, HiScript III RT SuperMix (Vazyme, R323‐01), and DEPC water was prepared. Complementary DNA (cDNA) was then synthesized using the SimpliAmp Thermal Cycler (Applied Biosystems, USA). Prepared DNA, primers, and SYBR Green dye (Vazyme, Q712‐02) were mixed in a specific ratio and transferred to an RNase‐free 96‐well plate. Real‐time fluorescence changes were recorded using a real‐time fluorescent quantitative PCR system (QuantStudio 5, Thermo, USA).

### 2.18 In Vivo Efficacy of Z‐H in Treating Infected Bone Defects

To assess the antibacterial and bone repair capabilities of Z‐H in vivo, we employed a rat tibial infected bone‐defect model. This animal study was approved by the Animal Research Committee of Tongji Medical College, Huazhong University of Science and Technology, Wuhan, China. Male Sprague–Dawley (SD) rats weighing between 220 and 250 g were randomly divided into four groups: Control (Ctrl), MRSA, MRSA+Z‐H (Z‐H), and MRSA+Z‐H+US (Z‐H/US). After anesthesia with 1% pentobarbital sodium, the tibial infected bone defect model was constructed in the following manner: The skin, subcutaneous fascia, and muscles were incised to expose the tibial plateau of the right hind leg, and a bone defect with a diameter of 3 mm was drilled using an electric drill. Subsequently, 100 µL of MRSA bacterial suspension (10^9^ CFU mL^−1^) was injected into the defect. After the bacterial suspension was fully absorbed, 300 µL of sterile PBS or Z‐H dispersion (500 µg mL^−1^) was injected into the defect site. Finally, the bone defect was sealed with bone wax to prevent fluid leakage, and the muscles and skin were sutured.

Post‐model establishment, rats in the Z‐H/US group received US treatment with the following parameters: intensity of 1.5 W cm^−2^, duty cycle of 50%, frequency of 1 MHz, and irradiation time of 15 min. On day 28 post‐modeling, all rats were euthanized, and photographs of the modeled area were taken. Tibial bone and muscle samples from the modeled site were collected from each rat and fixed in a 4% paraformaldehyde solution for 7 days. All tibial samples were scanned using a micro‐CT system (Bruker, Skyscan 1176) and analyzed using 3D reconstruction software to build 3D images. The tibial samples were then decalcified at room temperature for 30 days, sectioned, and subjected to H&E staining and IF staining for TNF‐*α*, OCN, and ALP.

### 2.19 In Vivo Efficacy of Z‐H in Treating Infected Skin Wounds

The experimental animals used were 6‐week‐old female KM mice, purchased from the Hubei Provincial Center for Disease Control and Prevention. This animal study protocol was approved by the Animal Research Committee of Tongji Medical College, Huazhong University of Science and Technology. The mice were randomly divided into four groups: Control (Ctrl), MRSA, Z‐H, and MRSA + Z‐H + US (Z‐H/US). Initially, the mice were anesthetized via intraperitoneal injection of 1% pentobarbital sodium (30 mg kg^−1^). Subsequently, the hair on the dorsal skin of the mice was shaved and disinfected, and a full‐thickness skin wound with a diameter of approximately 8 mm was created using a skin punch on the dorsal skin. In the MRSA and Z‐H/US groups, 100 µL of MRSA bacterial suspension (10^9^ CFU mL^−1^) was injected into the wound. After allowing the bacterial suspension to be fully absorbed, 300 µL of sterile PBS or Z‐H dispersion (500 µg mL^−1^) was injected into the wound site. Mice in the Z‐H/US group received US treatment (1.5 W cm^−2^, 50% duty cycle, 1 MHz, 15 min). Wound images were collected at specific time points (0, 3, 6, 9, and 12 days), and the wound area was calculated using ImageJ software. On day 12 post‐surgery, the mice were euthanized, and wound tissue samples as well as heart, liver, spleen, lung, and kidney tissues were collected. These tissues were fixed in a 4% paraformaldehyde solution, followed by paraffin embedding and sectioning. Sections of wound tissue from different groups were subjected to H&E staining, Masson's trichrome staining, and IF staining for inducible nitric oxide synthase (INOS), interleukin (IL‐6), CD31, and *α*‐SMA, as well as immunohistochemical staining for IL‐6 and TNF‐*α*. H&E staining was also performed on heart, liver, spleen, lung, and kidney tissues to explore the in vivo biosafety of Z‐H.

### 2.20 Treatment of Bacterial Biofilm‐Infected Skin Wounds With Z‐H@HA MNs

The experimental animals were 6‐week‐old female KM mice, purchased from the Hubei Provincial Center for Disease Prevention and Control. This animal study protocol was approved by the Animal Research Committee of Tongji Medical College, Huazhong University of Science and Technology. The mice were randomly divided into four groups: Control (Ctrl), MRSA, Z‐H@HA MN (Z‐H), and MRSA + Z‐H@HA MN + US (Z‐H/US). The method of creating skin wounds was the same as previously described. At the time of wound creation and two days post‐wounding, 100 µL of MRSA bacterial suspension (10^9^ CFU mL^−1^) was injected into the wounds of the MRSA and Z‐H/US groups to establish a wound biofilm infection. On the third day post‐wounding, Z‐H@HA MNs were inserted into the wounds of the Z‐H and Z‐H/US groups, and the Z‐H/US group was treated with US (1.5 W cm^−2^, 50% duty cycle, 1 MHz) for 15 min. Wound images were collected at postoperative days 0, 3, 6, 9, and 12, and wound area and healing rate were calculated using ImageJ software. On day 12 post‐surgery, the mice were euthanized, and wound tissue samples were collected and fixed in a 4% paraformaldehyde solution, followed by paraffin embedding and sectioning. Sections of wound tissue from different groups of mice were subjected to H&E staining, Masson's trichrome staining, and IF staining for INOS, IL‐6, CD31, and *α*‐SMA, as well as immunohistochemical staining for IL‐6 and TNF‐*α*.

### 2.21 Promotion of Skin Wound Healing by Z‐H@KGM‐XG Hydrogel

The experimental animals utilized in this study were 6‐week‐old female KM mice, sourced from the Hubei Provincial Center for Disease Prevention and Control. The animal experimental protocol was approved by the Animal Research Committee of Tongji Medical College, Huazhong University of Science and Technology. The mice were randomly assigned to three groups: Control (Ctrl), Z‐H@KGM‐XG Hydrogel (Z‐H), and Z‐H@KGM‐XG Hydrogel+US (Z‐H/US). The method for creating skin wounds was consistent with previous methods described. Post‐wounding, the Z‐H@KGM‐XG Hydrogel was applied to cover the wounds of the Z‐H and Z‐H/US groups, and the Z‐H/US group was treated with US (1.5 W cm^−2^, 50% duty cycle, 1 MHz) for 15 min. Wound images were collected on postoperative days 0, 3, 6, 9, and 12, and the wound area and healing rate were calculated using ImageJ software. On the 12th postoperative day, the mice were anesthetized via intraperitoneal injection of 1% pentobarbital sodium (30 mg kg^−1^), and wound tissue samples from each group were harvested for RNA sequencing analysis.

### 2.22 Statistical Analysis

Data were processed in GraphPad Prism 9 and Origin 2021. Results are reported as mean ± standard deviation from a minimum of three independent replicates. Depending on the comparison, statistical significance was assessed with Student's t‐test, one‐way ANOVA, or two‐way ANOVA. Significance thresholds were set at *p < 0.05, **p < 0.01, ***p < 0.001, and ****p < 0.0001.

## 3 Results and Discussion

### 3.1 Characterization of Z‐H Nanoparticles

**FIGURE 1:**
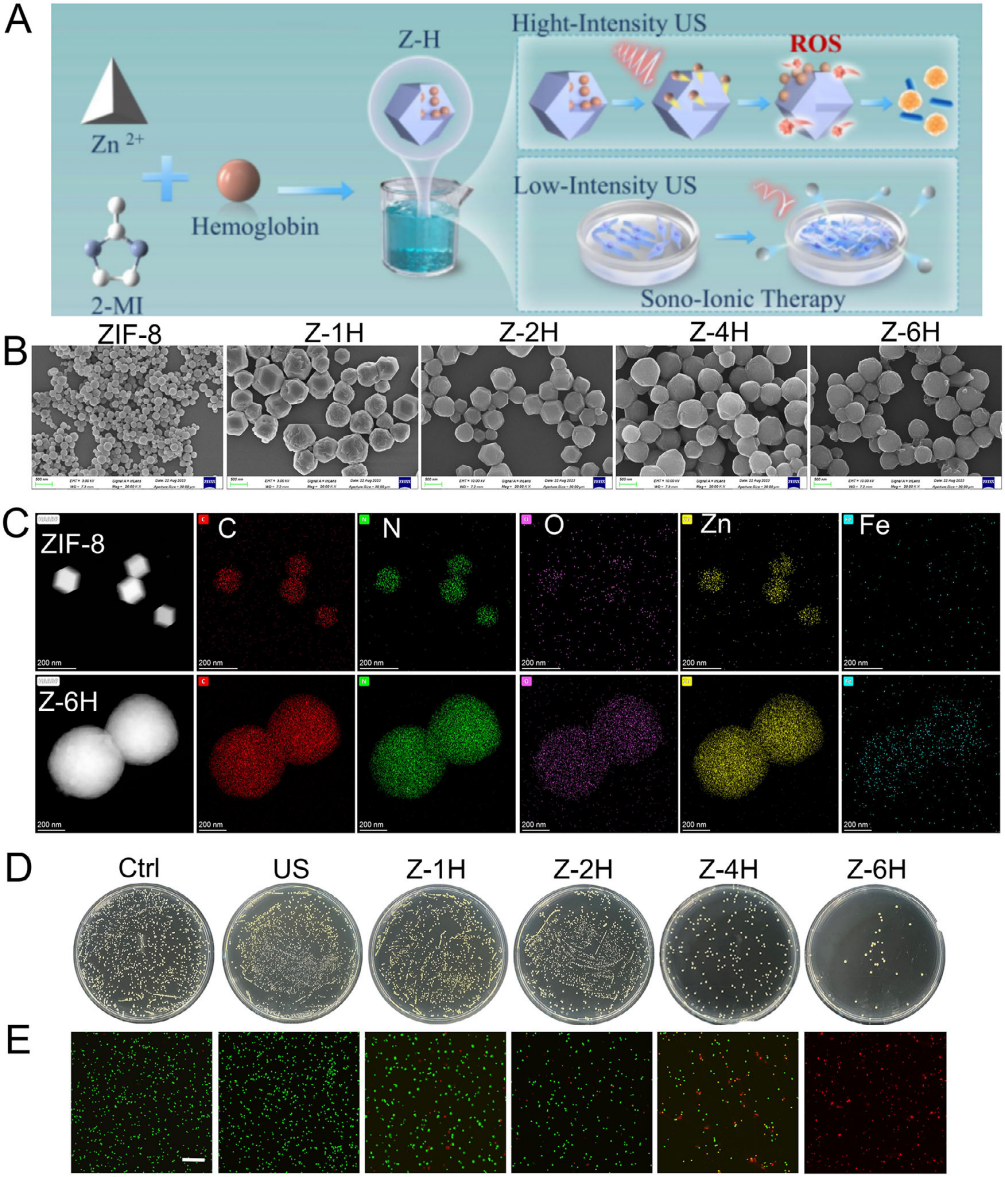
Fabrication process, morphology characterization, and antibacterial performance. (A) Schematic illustration of the preparation procedure of different nanoparticles. (B) SEM images of different nanoparticles. Scar bar: 500 nm. (C) TEM images and elemental mapping of ZIF‐8 and Z‐6H. Scar bar: 200 nm. (D) Spread plate images of MRSA of the different nanoparticles with or without US irradiation (1.5 W cm^−2^, 50% duty cycle,1 MHz) for 15 min. (E) Live/dead staining of MRSA of the different nanoparticles with or without US irradiation (1.5 W cm^−2^, 50% duty cycle,1 MHz) for 15 min. Scar bar: 50 µm.

Figure 1A schematically illustrates the fabrication process of ZIF‐8 and different Z‐H nanoparticles through the incorporation of Hb into the ZIF‐8 framework. The SEM images in Figure 1B demonstrate the successful synthesis of different Z‐H nanoparticles, and the different groups have uniform morphological characteristics. The average diameter of the ZIF‐8 was approximately 200 nm (Figure S1B), and after inducing Hb, the average diameter was increased (Figure S1C), indicating the effect of Hb. The elemental mapping analysis presented in Figure 1C and Figure S1A demonstrates the spatial distribution of C, N, O, Zn, and Fe elements within the nanoparticles. The homogeneous distribution of Fe elements throughout the Z‐H nanoparticles provides strong evidence for the successful encapsulation of Hb within the ZIF‐8. Quantitatively, UV–Vis standard‐curve analysis yielded Hb LCs of 0.0% (ZIF‐8), 4.1% (Z‐1H), 7.7% (Z‐2H), 10.8% (Z‐4H), and 12.9% (Z‐6H), with corresponding EEs of 0.0%, 68.2%, 63.3%, 58.5%, and 52.8%, respectively.

To further validate the ion‐release behavior, we quantified time‐resolved Zn^2+^ liberation from Z‐H by ICP under identical pH and temperature with or without low‐intensity US. As shown in Figure S2, US irradiation accelerated Zn^2+^ release, leading to consistently higher cumulative concentrations at each time point and a faster approach to plateau compared with the non‐US control. These data indicate that acoustic stimulation facilitates Zn^2+^ out‐diffusion/erosion of the ZIF‐8 framework and support the proposed sono‐ionic contribution that cooperates with Hb‐mediated sonodynamic effects.

### 3.2 In Vitro Antibacterial Activity of Z‐H Nanoparticles

To evaluate the antibacterial capability of Z‐H nanoparticles, we conducted systematic in vitro antibacterial experiments. Figure 1D presents the results of the antibacterial assays for various Z‐H nanoparticles under US irradiation, demonstrating that Z‐6H nanoparticles exhibit the highest antibacterial efficiency. Additionally, the results presented in Figures S1D and S1E indicate that in the absence of US irradiation, neither ZIF‐8, Hb, nor the Z‐H groups exhibited significant antibacterial effects. This finding was further corroborated by the bacterial viability staining results shown in Figure 1E, where the live/dead fluorescence images revealed that Z‐6H nanoparticles achieved superior bacterial eradication efficiency under US stimulation. The remarkable antibacterial performance of Z‐6H nanoparticles may be attributed to their optimal structural characteristics and enhanced sonocatalytic performance to ROS. Based on these compelling results, Z‐6H nanoparticles were selected for subsequent cellular experiments and in vivo studies.

### 3.3 Electrochemical Characterization and ROS Generation Detection

**FIGURE 2:**
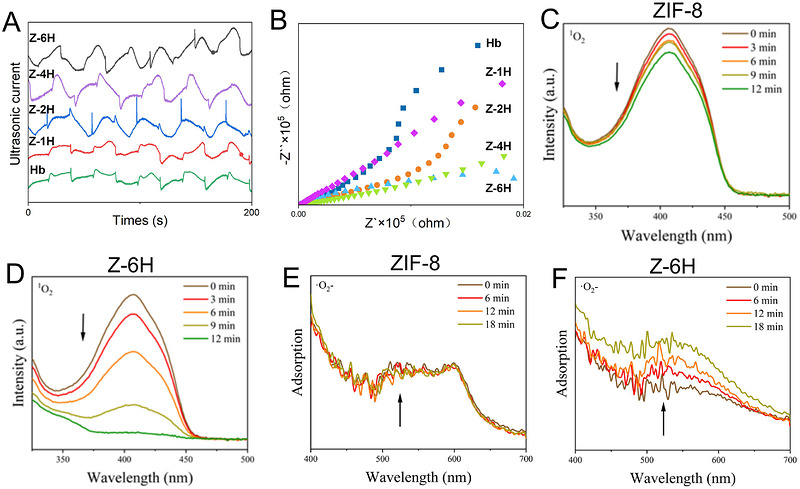
Sonocatalytic detection and ROS generation ability. (A) Sonocurrent test of different nanoparticles. (B) Electrochemical impedance measurement of different nanoparticles. (C, D) The ^1^O_2_ generation of ZIF‐8 and Z‐6H under US detecting by the decrease of DMA fluorescence intensity. (E, F) Adsorption of NBT treated by ZIF‐8 and Z‐6H for 18 min under US.

To further investigate the sonocatalytic activity of different Z‐H nanoparticles, we systematically evaluated their electrochemical properties using an electrochemical workstation. As shown in Figure 2A, the ultrasonic current response measurements revealed that Z‐H nanoparticles, particularly Z‐6H, exhibited significantly enhanced current generation under US irradiation compared to others. This remarkable current response suggests that Z‐H materials can effectively convert ultrasonic energy into electrical energy to produce ROS. The EIS results (Figure 2B) further confirmed the superior electron transfer capability of Z‐H materials, with Z‐6H demonstrating the smallest semicircular arc, indicating the lowest charge transfer resistance. This characteristic implies that Z‐6H can facilitate more efficient electron transfer under US irradiation, thereby enhancing its sonocatalytic activity.

To assess the ROS generation capacity of each sample under US conditions, we employed DPBF and NBT as specific probes for singlet oxygen (^1^O_2_) and superoxide anion (•O_2_^−^) detection, respectively. The results demonstrated a significant enhancement in ^1^O_2_ generation by Z‐H materials under US irradiation, with Z‐6H showing the most pronounced ^1^O_2_ production (Figure 2C,D and Figure S3A–C). Similarly, Z‐6H exhibited the strongest •O_2_^−^ generation capability among all tested samples (Figure 2E,F and Figure S3D–F). However, no generation of ^1^O_2_ or •O_2_^−^ was detected in the US‐only group without Z‐H nanoparticles (Figure S3G–H). These findings collectively indicate that Z‐6H can efficiently generate both ^1^O_2_ and •O_2_^−^ under US stimulation, which represents the fundamental mechanism underlying its bactericidal activity. The generated ROS can effectively disrupt bacterial cell membranes, ultimately leading to bacterial inactivation.

### 3.4 Cytotoxicity and Osteogenic Differentiation Ability of Z‐H in Vitro

**FIGURE 3:**
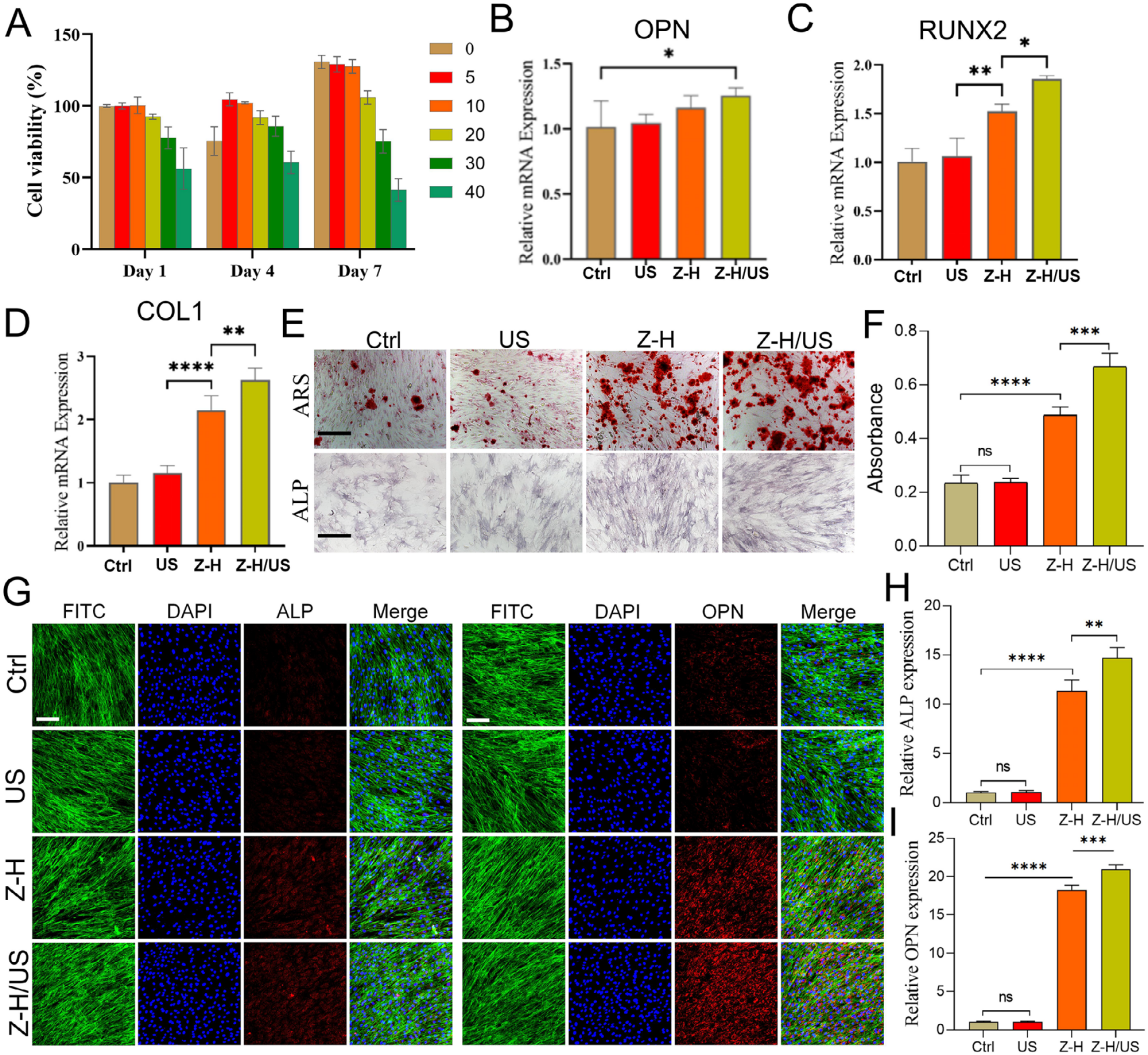
Cell viability and in vitro osteogenesis performance. (A) The cytocompatibility of Z‐H detecting by CCK‐8 assay. (B, C, D) OPN, RUNX2, COL1 qRT‐PCR results of hBMSCs treated in different conditions (control, US, Z‐H, and Z‐H +US) after 14 days. (E, F) ALP and ARS staining images of hBMSCs cultured in different conditions after 21 days and quantitative analysis of ARS. Scar bar: 50 µm. (G) ALP and OPN immunofluorescence staining images of hBMSCs cultured in different conditions after 21 days (green fluorescence: cytoskeleton; blue fluorescence: nucleus; red fluorescence: ALP or OPN). Scar bar: 50 µm. Quantitative analysis of (H) ALP and (I) OPN fluorescence intensity after 21 days. *n* = 3 independent experiments per group, *p < 0.05, **p < 0.01, ****p < 0.0001, ns = not significant.

To investigate the cytotoxicity of Z‐H and osteogenic differentiation ability under US intervention, we conducted a series of in vitro cellular experiments using hBMSCs. The CCK‐8 assay (Figure 3A) revealed that Z‐H nanoparticles at concentrations up to 10 µg mL^−1^ maintained excellent cell viability, demonstrating favorable biocompatibility within this concentration range. However, a notable decrease in cell viability was observed at concentrations exceeding 10 µg mL^−1^, indicating dose‐dependent cytotoxicity. Based on these findings, 10 µg mL^−1^ was selected as the optimal concentration for subsequent cellular osteogenic differentiation experiments. qPCR analysis of osteogenic marker genes demonstrated significant upregulation of OPN (Figure 3B), RUNX2 (Figure 3C), and COL1 (Figure 3D) mRNA expression levels in hBMSCs treated with 10 µg mL^−1^ Z‐H nanoparticles with or without US. Notably, the combination of Z‐H with US intervention (Z‐H/US group) resulted in further enhancement of these gene expressions. These molecular markers, crucial for osteogenic differentiation, suggest that Z‐H nanoparticles effectively promote hBMSCs differentiation toward osteogenic lineage, with US intervention providing additional synergistic effects. ARS and ALP staining (Figure 3E) further revealed significantly increasing mineralization nodule formation and ALP activity in Z‐H‐treated groups compared to controls. The Z‐H/US group exhibited the most pronounced effects, with both mineralization and ALP activity surpassing those observed in the Z‐H group alone. Quantitative analysis of ALP staining (Figure 3F) confirmed these observations, showing significantly higher ALP activity in the Z‐H/US group compared to both Z‐H and US groups. IF staining and quantitative analysis of ALP and OPN protein expression (Figure 3G–I) provided additional evidence supporting the enhanced osteogenic potential of the Z‐H/US combination. The Z‐H/US group demonstrated significantly higher expression levels of both osteogenic markers compared to other experimental groups. The observed osteogenic effects may be attributed to multiple mechanisms. The zinc ions released from Z‐H nanoparticles potentially activate various signaling pathways that promote osteoblast proliferation and differentiation. Additionally, the sonosensitive properties of Z‐H materials, when combined with low‐intensity US intervention, generate localized microcurrents that may further enhance osteogenic differentiation. These microcurrents potentially modulate intracellular calcium ion concentration, subsequently influencing the expression of osteogenesis‐related genes.

### 3.5 Therapeutic Efficacy of Z‐H in Rat‐Infected Bone Defects

**FIGURE 4:**
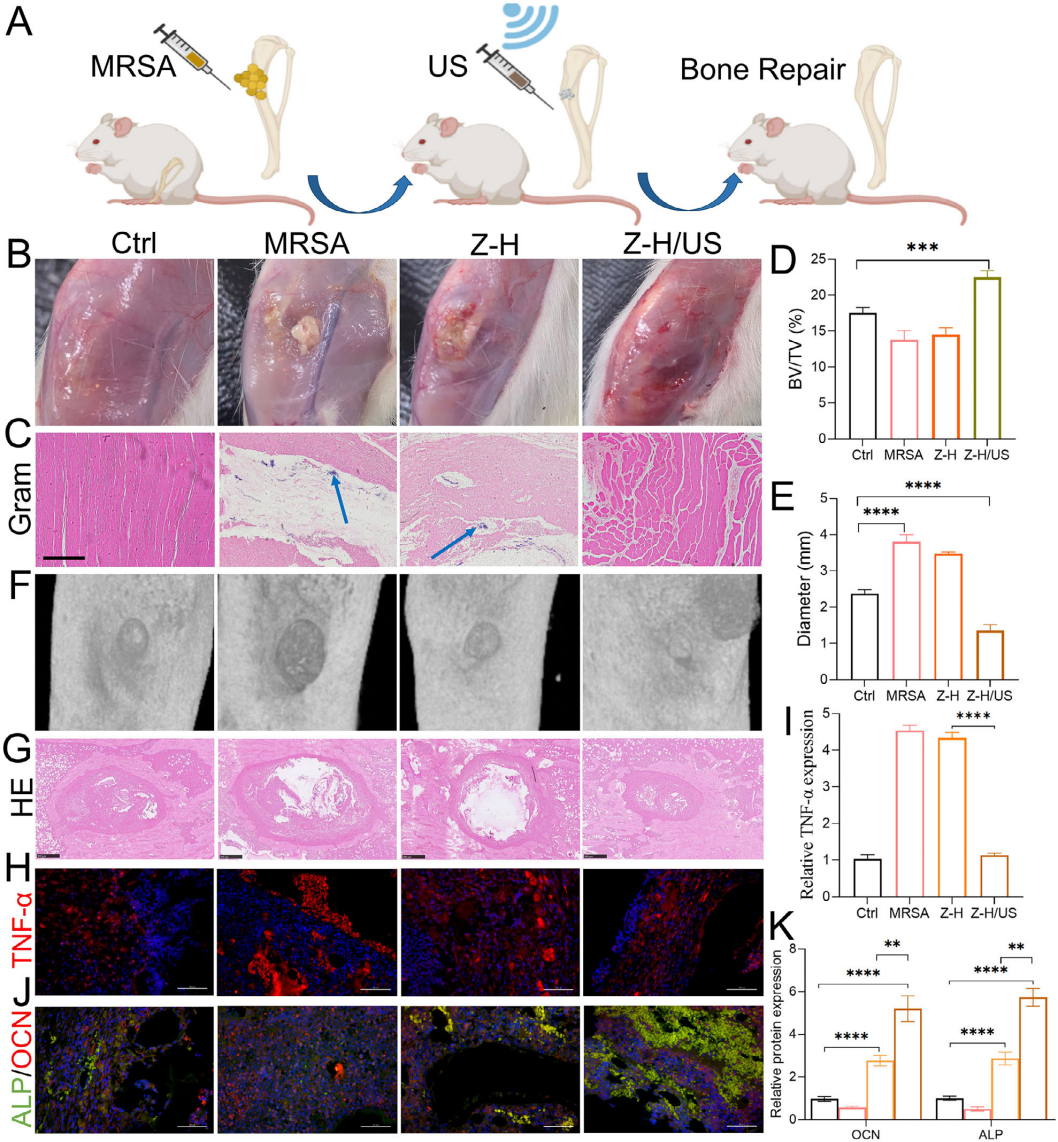
Treatment of osteomyelitis. (A) Schematic diagram of sonodynamic therapy for osteomyelitis using Z‐H in vivo. (B) Images of the surgical site of the infected leg. (C) Gram stain images of muscle tissue near the sites of infection. MRSA are highlighted with red arrows. Scar bar: 500 µm. (D) Quantified BV/TV results of bone defects. (E) Quantitative statistics of bone defect diameter. (F) Micro‐CT analysis and reconstruction results. (G) HE staining images of infected bone. (H) Immunofluorescence of bone tissue on Day 28 using TNF‐*α* staining (Scale bar = 50 µm) and the corresponding fluorescence intensity (I). (J) Immunofluorescence of bone tissue on Day 28 using ALP and OCN staining (Scale bar = 50 µm) and the corresponding fluorescence intensity (K). *n* = 3 independent experiments per group, **p < 0.01, ***p < 0.001, ****p < 0.0001.

To evaluate the anti‐infective and tissue regeneration capabilities of Z‐H nanoparticles in vivo, we established a rat model of a tibial infected bone defect and utilized varying intensities of US to achieve early‐stage high‐intensity US for ROS generation to eliminate bacteria and later‐stage low‐intensity US for the production of microcurrents to facilitate sono‐ionic therapy to promote tissue repair. The experimental design and workflow are illustrated in Figure 4A. At 28 days post‐modeling, macroscopic examination of the modeled sites (Figure 4B) revealed significantly reduced infection symptoms in the Z‐H/US group. Gram staining of muscle tissues surrounding the defect sites (Figure 4C) further confirmed the enhanced antibacterial efficacy of Z‐H under US intervention, with the Z‐H/US group showing substantially reduced bacterial colonization compared to other groups. To evaluate the bone repair ability, Micro‐CT analysis of bone defect sites demonstrated significant improvements in bone regeneration parameters. The BV/TV ratio (Figure 4D) was significantly higher in the Z‐H/US group compared to both control and MRSA‐infected groups, indicating enhanced bone defect repair. Notably, the Z‐H group alone showed limited bone regeneration, which can be attributed to the lack of antibacterial activity in the absence of US, allowing bacterial infection to compromise bone tissue repair. Quantitative analysis of bone defect diameter (Figure 4E) supported these findings, with the Z‐H/US group exhibiting the smallest defect diameter among all groups. Representative 3D reconstructed micro‐CT images (Figure 4F) visually confirmed the superior bone regeneration in the Z‐H/US group, showing a substantial reduction in defect area compared to other groups. Histological examination through H&E staining (Figure 4G) provided further evidence of enhanced bone regeneration in the Z‐H/US group, demonstrating a more organized bone structure with increased trabecular number and density. To evaluate the inflammatory response and expression of osteogenic protein in vivo, IF analysis revealed significant biological responses at the molecular level; TNF‐*α* expression (Figure 4H) was markedly reduced in the Z‐H/US group compared to the MRSA and Z‐H groups, indicating effective MRSA eradication and subsequent inflammation reduction under US intervention. Conversely, ALP and OCN expression levels (Figure 4J) were significantly elevated in the Z‐H/US group, suggesting enhanced osteogenic activity. Quantitative fluorescence analysis (Figure 4I,K) provided statistical validation of these observations, offering compelling evidence for the dual functionality of Z‐H nanoparticles in combating infection and promoting bone regeneration when combined with US therapy.

### 3.6 Therapeutic Efficacy of Z‐H in MRSA‐Infected Skin Wounds

**FIGURE 5:**
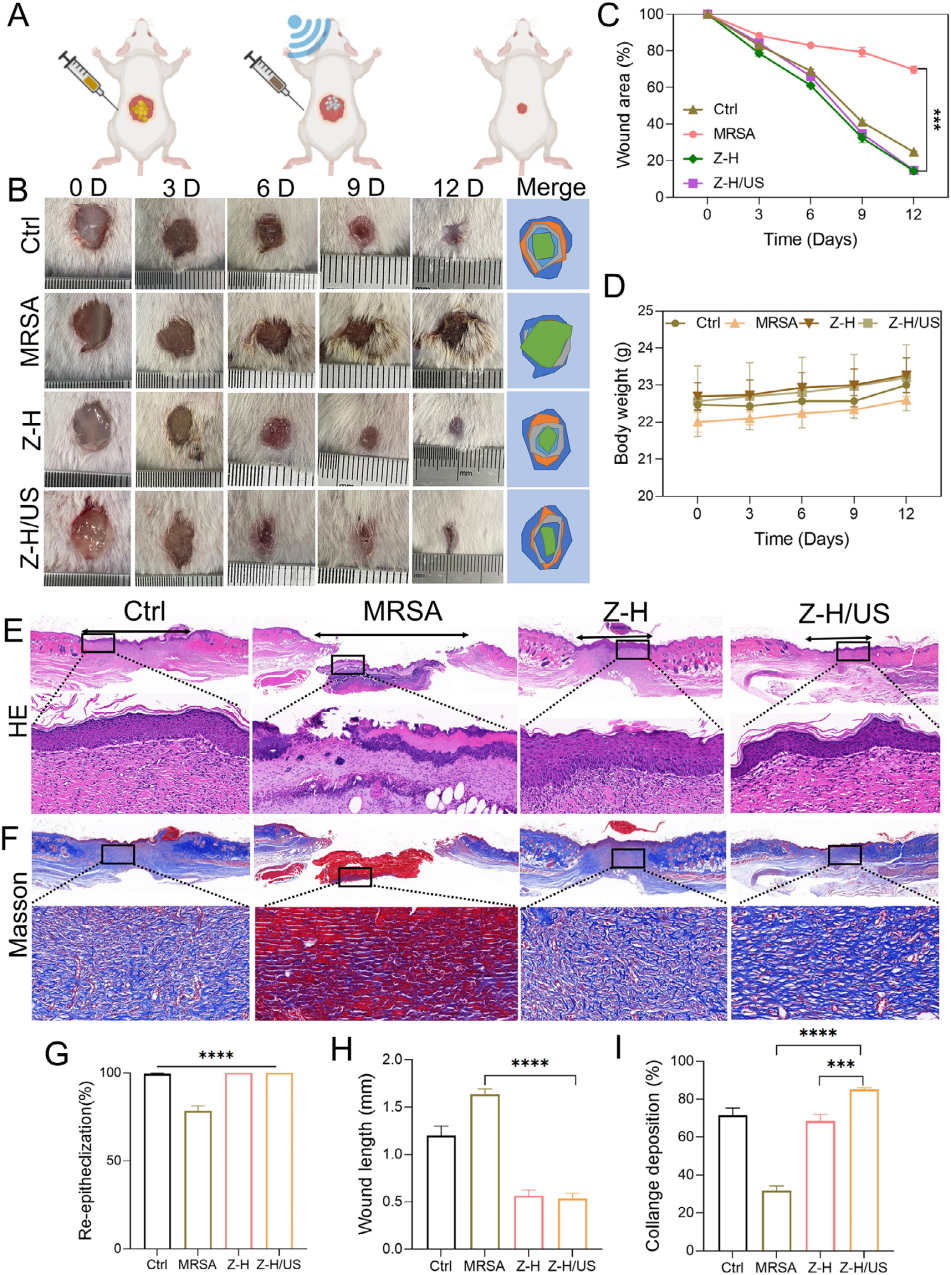
In vivo sonodynamic therapy for MRSA wound infections. (A) Scheme of sonodynamic therapy of Z‐H for MRSA wound infections in vivo. (B, C) Representative photographs of the wound healing process and corresponding quantitative analysis of wounds over time. (D) Changes of body weight over time after different interventions. (E, F) H&E and Masson staining of wound tissue for different groups after 14 days treatment (Scale bar = 500 and 100 µm). (G, H, I) Statistic analysis of re‐epitheclization, wound length, and collagen content between different groups. *n* = 3 independent experiments per group, ***p < 0.001, and ****p < 0.0001.

To evaluate the anti‐infective capabilities and wound healing of Z‐H nanoparticles in vivo, we established a mouse dorsal‐infected skin wound model. The experimental design and workflow are illustrated in Figure 5A. We continued to utilize high‐intensity US in the early stage to generate a significant amount of ROS to eliminate bacteria, followed by the application of low‐intensity US to produce microcurrents combined with Zn^2+^ ions to promote wound healing through sono‐ionic therapy. Wound healing progression was monitored at different time points (0, 3, 6, 9, and 12 days) post‐injury, with photographic documentation (Figure 5B) demonstrating accelerated wound closure in the Z‐H/US group, showing nearly complete healing by the end of the observation period. In contrast, the MRSA group exhibited delayed healing with persistent infection signs. Quantitative analysis of wound closure rates (Figure 5C) revealed significantly faster healing in both Z‐H and Z‐H/US groups compared to other groups. Body weight monitoring (Figure 5D) confirmed that all experimental procedures were well‐tolerated, with no significant weight fluctuations observed across groups.

Histopathological examination provided detailed insights into the healing process. H&E staining (Figure 5E) demonstrated more organized tissue architecture and reduced inflammatory cell infiltration in the Z‐H/US group, indicating effective inflammation control and tissue regeneration. Masson's trichrome staining (Figure 5F) further confirmed enhanced collagen deposition in the Z‐H/US group, suggesting improved extracellular matrix remodeling. Quantitative analysis revealed significant improvements in re‐epithelialization rates (Figure 5G), wound diameter reduction (Figure 5H), and collagen deposition (Figure 5I) in Z‐H‐treated groups, with the Z‐H/US combination showing the most pronounced effects.

**FIGURE 6:**
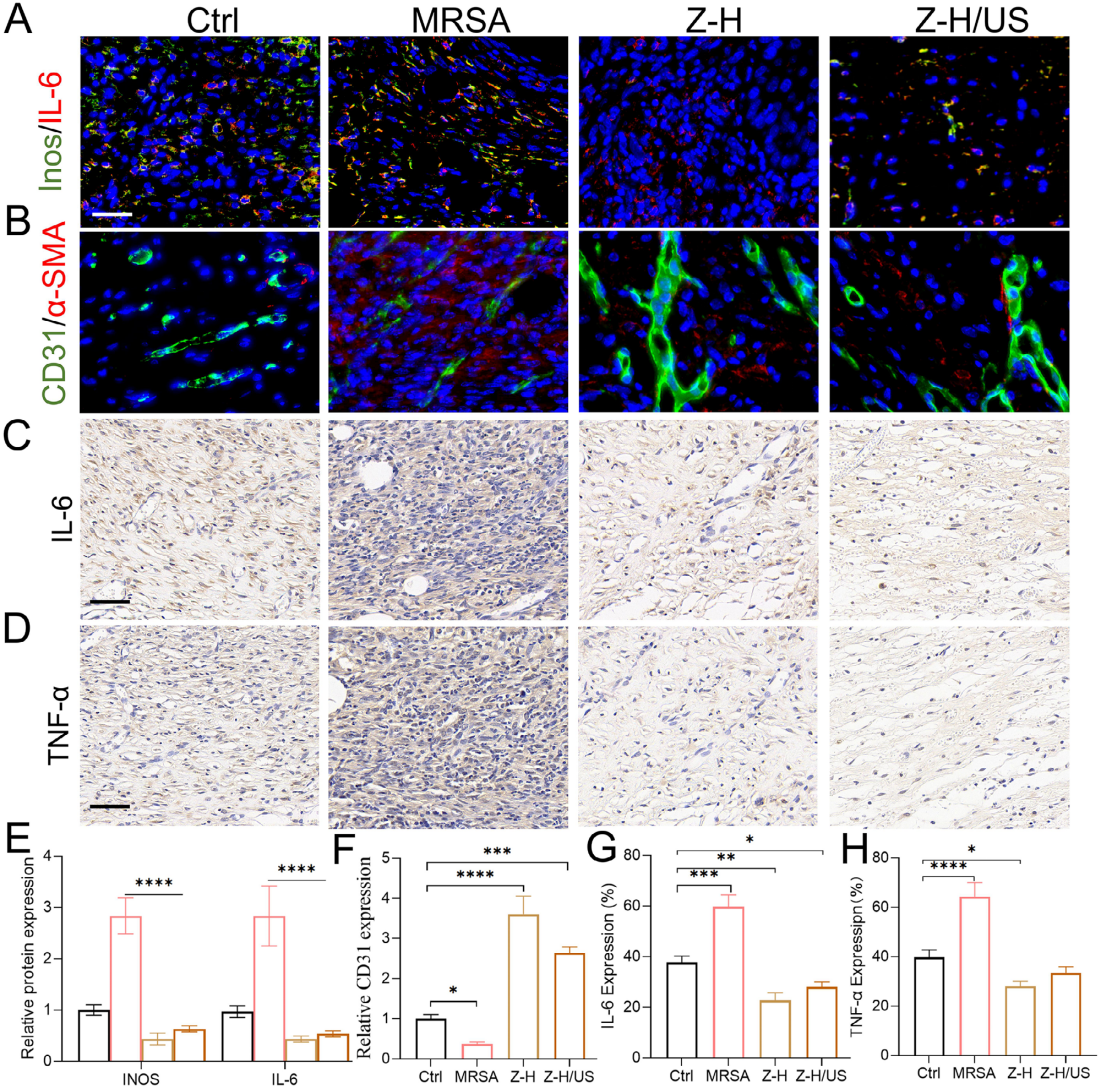
Histological evaluation of would tissue. (A) Immunofluorescence staining of inos and IL6 of wound tissue on Day14 (Scale bar = 50 µm) and the corresponding fluorescence intensity (E). (B) Immunofluorescence staining of CD31 and *α*‐SAM of wound tissue on Day14 (Scale bar = 50 µm) and the corresponding fluorescence intensity (F). (C, D) Immunohistochemical staining of IL‐6 and TNF‐*α*, and statistical analysis (G, H), *n* = 3 independent experiments per group, *p < 0.05, **p < 0.01, ***p < 0.001, ****p < 0.0001.

To elucidate the underlying mechanisms, we investigated the local inflammatory response and angiogenesis through IF and immunohistochemical staining. Analysis of inflammatory markers (Figure 6A,C,D) revealed significant downregulation of iNOS (Figure 6E), IL‐6 (Figure 6G), and TNF‐*α* (Figure 6H) expression in the Z‐H/US group compared to MRSA controls, demonstrating potent anti‐inflammatory effects. Angiogenesis assessment through CD31 and α‐SMA IF staining (Figure 6B) showed significantly enhanced CD31 expression in the Z‐H/US group (Figure 6F), indicating improved vascularization crucial for wound healing. Histopathological examination of major organs (Figure S4) revealed no apparent pathological changes, confirming the biosafety of both Z‐H nanoparticles and US treatment. However, this evaluation was limited to short‐term exposure; long‐term systemic toxicity, including blood chemistry, immunological response, and potential cumulative effects at higher or repeated doses, remains to be elucidated and will be a key focus of our future investigations.

These comprehensive results demonstrate that Z‐H nanoparticles, particularly when combined with US intervention, exhibit multifunctional therapeutic effects: (1) Effective MRSA eradication and modulation of inflammatory responses through enhanced antibacterial activity; (2) accelerated wound healing via improved re‐epithelialization and collagen deposition; and (3) promotion of angiogenesis to support tissue regeneration. The therapeutic efficacy may be attributed to the synergistic effects of US‐induced ROS generation, zinc ion release, and microcurrent stimulation, which collectively contribute to bacterial clearance and tissue repair processes.

### 3.7 Therapeutic Efficacy of Z‐H@HA MN in MRSA Biofilm‐Infected Skin Wounds

**FIGURE 7:**
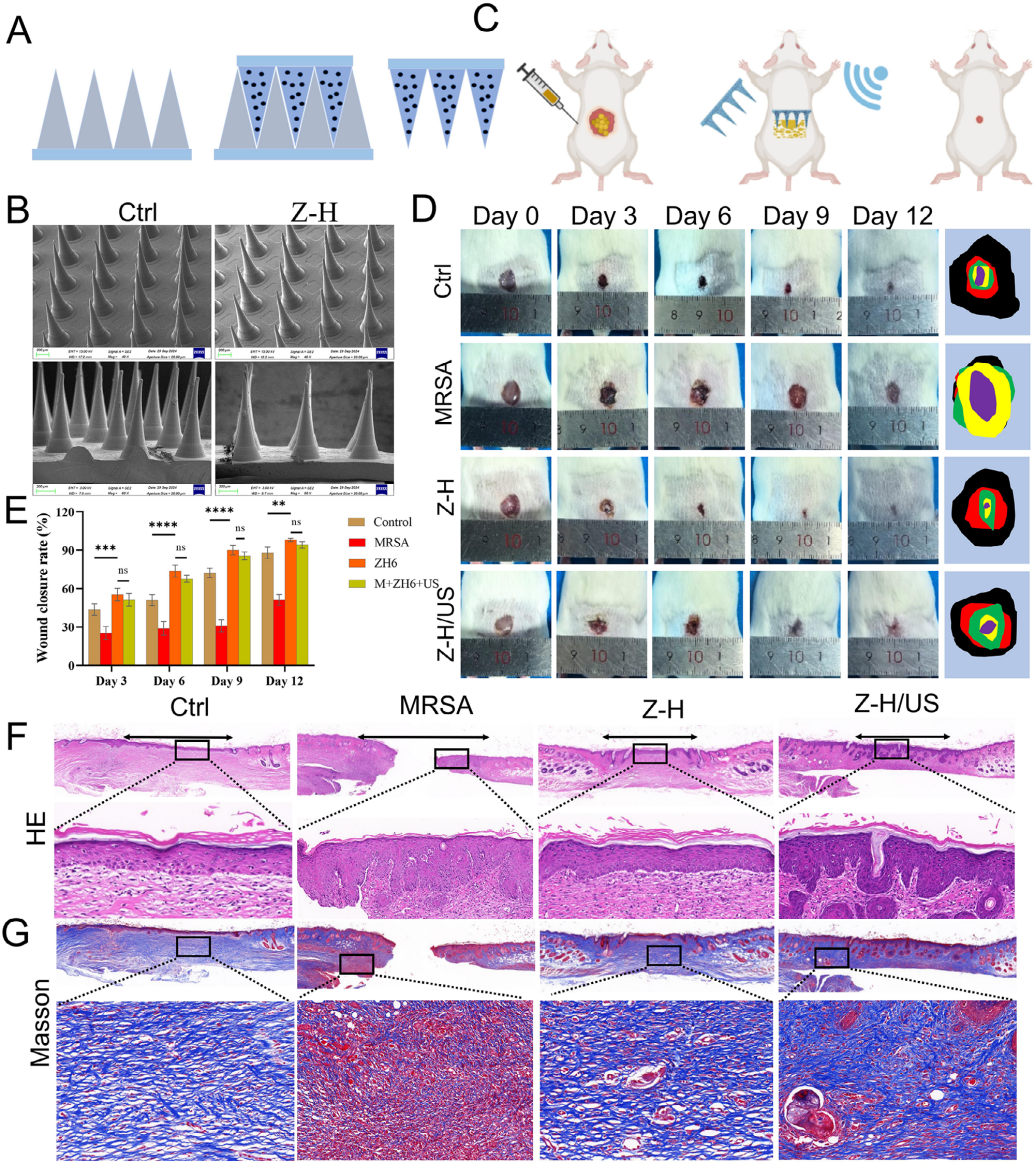
In vivo treatment of biofilm infection. (A) Microneedle preparation process with Z‐H. (B) SEM images of blank and Z‐H MNs. (C) Scheme of sonodynamic therapy of Z‐H MNs for MRSA biofilm wound infections in vivo. (D, E) Representative photographs of the wound healing process and corresponding quantitative analysis of wounds over time. (F, G) H&E and Masson staining of wound tissue for different groups after 14 days treatment (Scale bar = 500 and 100 µm). *n* = 3 independent experiments per group, *p < 0.05, **p < 0.01, ***p < 0.001, ****p < 0.0001.

If bacteria are not eliminated early, they can rapidly form biofilms, which not only protect the bacteria from drug treatments but are also located deep within the tissue. To evaluate the anti‐biofilm and wound healing capabilities of Z‐H, as shown in Figure 7A, we prepared HA MN and loaded Z‐H into the microneedles (Z‐H@HA), and we established a mouse model of MRSA biofilm‐infected skin wounds. The experimental design and workflow are illustrated in Figure 7C. SEM analysis (Figure 7B) confirmed the successful fabrication of Z‐H@HA MN, demonstrating well‐preserved microneedle architecture. In addition, crystal violet staining was conducted to evaluate the in vitro anti‐biofilm performance of Z‐H@HA MN under US (Figure S5A). The results showed that only the Z‐H MN + US group exhibited significant biofilm disruption, whereas the MN alone or without US had negligible effects. Wound healing progression analysis revealed significant therapeutic benefits of Z‐H@HA MN treatment. Photographic documentation (Figure 7D) and quantitative wound closure assessment (Figure 7E) demonstrated accelerated healing in both Z‐H without MRSA (Z‐H@HA MN) and Z‐H/US (MRSA+Z‐H@HA MN+US) with MRSA groups compared to control and MRSA groups. The histopathological examination provided detailed insights into the therapeutic mechanisms. H&E staining (Figure 7F) revealed more organized tissue architecture and reduced inflammatory cell infiltration in Z‐H‐treated groups, particularly in the Z‐H/US group. Masson's trichrome staining (Figure 7G) further confirmed enhanced collagen deposition and extracellular matrix remodeling in Z‐H‐treated wounds, suggesting improved tissue regeneration. IF (Figure S5B,C) and immunohistochemical staining (Figure S5D,E) analyses provided molecular‐level evidence of the therapeutic effects. The Z‐H/US group exhibited significant downregulation of inflammatory markers (iNOS, IL‐6, and TNF‐*α*) compared to the MRSA group, indicating effective inflammation control. Concurrently, enhanced expression of angiogenesis markers (CD31 and *α*‐SMA) in the Z‐H/US group suggested improved vascularization, which is crucial for wound healing.

These comprehensive results demonstrate that Z‐H@HA MN, particularly when combined with US intervention, exhibits multifunctional therapeutic capabilities: (1) Effective disruption and eradication of MRSA biofilms; (2) accelerated wound healing through enhanced tissue regeneration; and (3) promotion of angiogenesis to support tissue repair. The therapeutic efficacy can be attributed to the synergistic effects of Z‐H nanoparticles and HA microneedles, which facilitate targeted delivery of antibacterial agents and sustained release of therapeutic components, combined with the enhanced penetration and antibacterial activity provided by US intervention.

### 3.8 Therapeutic Mechanisms of Z‐H Under US Therapy

**FIGURE 8:**
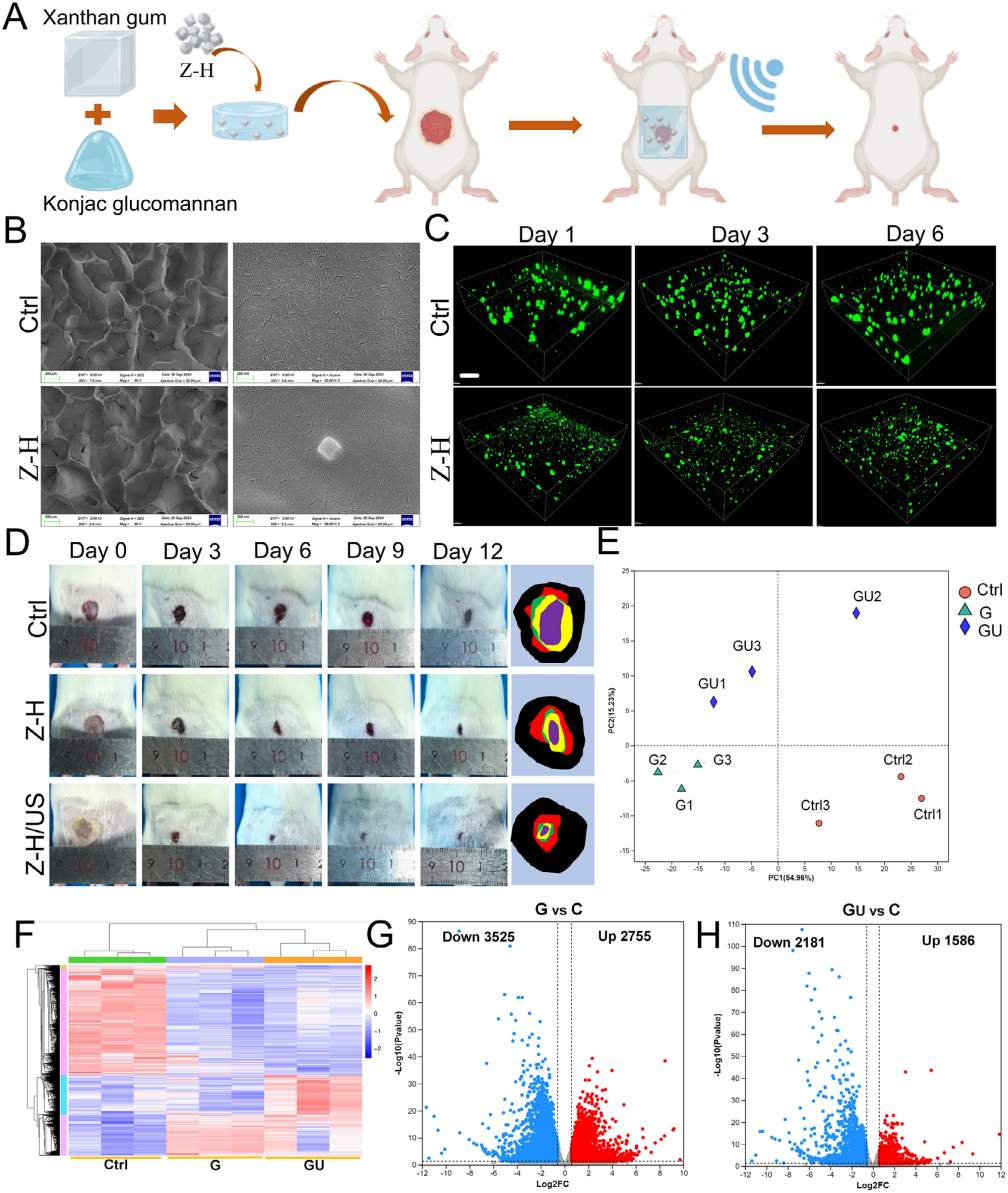
Therapeutic mechanisms of Z‐H under US therapy. (A) Preparation process of KGM‐XG hydrogel with Z‐H. (B) SEM images of KGM‐XG hydrogel with or without Z‐H. (C) Cell activity of Z‐H hydrogel over time. Scar bar: 200 µm. (D) Representative photographs of the wound healing process. (E) Principal component analysis (PCA) between different groups. (F) Heatmap showing differentially expressed genes (DEGs) between each group. (G) Volcano plot of G vs C and GU vs C. *n* = 3 independent experiments per group.

To investigate the wound healing efficacy and underlying biological mechanisms of Z‐H under US with low intensity, we prepared a KGM‐XG hybrid hydrogel with excellent biosafety and water retention properties to load Z‐H, aiming to investigate its mechanism in tissue repair (Figure 8A). We established a mouse skin wound model and conducted comprehensive transcriptomic analysis. SEM imaging of lyophilized hydrogels (Figure 8B) revealed a well‐defined porous structure in both KGM‐XG and Z‐H@KGM‐XG hydrogels, with Z‐H nanoparticles uniformly distributed throughout the KGM‐XG matrix, confirming successful loading. This hierarchical architecture not only facilitates cell attachment and proliferation but also ensures sustained release of therapeutic components. Live/dead staining of hBMSCs cultured with the hydrogels (Figure 8C) demonstrated excellent compatibility of Z‐H@KGM‐XG Hydrogel throughout the observation period. In addition, the practical dressing attributes of both hydrogels were quantified. Swelling‐ratio tests (Figure S6B) revealed that KGM‐XG and Z‐H@KGM‐XG gels absorbed fluid rapidly during the first 12 h and reached equilibrium at ≈150% of their original weight within 80 h, with no significant difference between the two formulations. This high yet stable water uptake is desirable for maintaining a moist wound environment while preventing maceration. Compression studies further showed that embedding Z‐H nanoparticles enhanced mechanical robustness: the Z‐H@KGM‐XG gel withstood ∼70 kPa at 35% strain versus ∼30 kPa for the pristine gel (Figure S6C), and both materials retained their stress–strain profiles over repeated loading–unloading cycles (Figure S6D), indicating good elasticity and fatigue resistance. Together, the swelling and mechanical data confirm that Z‐H@KGM‐XG Hydrogel possesses the absorptive capacity and structural integrity needed for clinical wound‐dressing applications while simultaneously delivering therapeutic nanoparticles.

**FIGURE 9:**
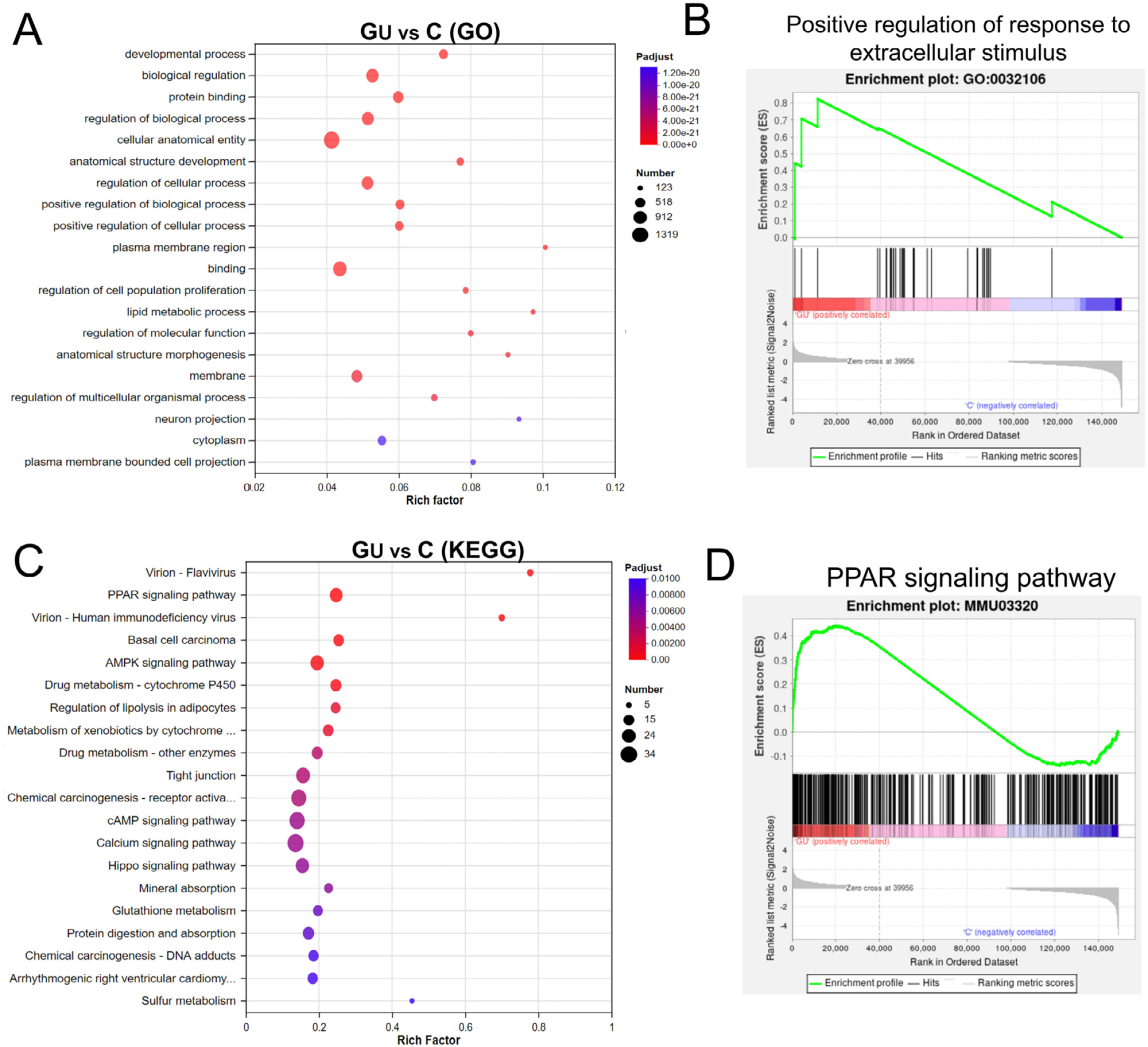
Function analysis. (A) GO enrichment analysis of GU vs C. (B) KEGG enrichment analysis of GU vs C. (C, D) GSEA analysis of differentially expressed genes (DEGs). Plots are relative to positive regulation of response to extracellular stimulus and PPAR signaling pathway. *n* = 3 independent experiments per group.

Wound healing progression analysis showed significant therapeutic benefits of Z‐H@KGM‐XG Hydrogel treatment. Photographic documentation (Figure 8D) and quantitative wound closure assessment (Figure S6A) demonstrated accelerated healing in both Z‐H and Z‐H/US groups, with the Z‐H/US combination showing the most pronounced effects, indicating synergistic enhancement of wound repair by US intervention. We performed RNA sequencing analysis of wound tissues collected at day 12 post‐treatment to elucidate the molecular mechanisms underlying the enhanced wound healing. Principal component analysis (PCA) (Figure 8E) revealed distinct clustering patterns among treatment groups, indicating significant transcriptomic alterations. Heatmap analysis of differentially expressed genes (DEGs) (Figure 8F) and statistical analysis of DEG numbers (Figure S7A) demonstrated comprehensive gene expression changes. Volcano plot analysis of DEGs between KGM‐XG (G) vs control (C), Z‐H/US (GU) vs control (Figure 8G,H), and Z‐H/US vs Z‐H (Figure S7B) provided detailed insights into transcriptional regulation. Functional annotation analysis revealed significant biological processes and signaling pathways associated with enhanced wound healing. Gene ontology (GO) enrichment analysis of GU vs C (Figure 9A) and U vs C (Figure S8A) identified several significantly enriched biological processes, including “developmental process,” “biological regulation,” and “protein binding,” with particular emphasis on “positive regulation of response to extracellular stimulus” (Figure 9B), suggesting that Z‐H@KGM‐XG Hydrogel under US irradiation might promote wound healing through enhanced cellular response to external stimuli. Kyoto Encyclopedia of Genes and Genomes (KEGG) pathway analysis of U vs C (Figure S8B) showed that the Hippo signaling pathway, Wnt signaling pathway, and cell cycle play an important role in wound regeneration for KGM‐XG Hydrogel. Figure 9C further revealed significant enrichment of several signaling pathways, particularly the PPAR signaling pathway (Figure 9D), which plays crucial roles in the wound healing process with Z‐H after US irradiation. To confirm that the up‐regulation observed in the RNA‐seq analysis indeed reflects activation of the PPAR signaling cascade, we further quantified the mRNA levels of four canonical PPAR‐regulated genes—PPAR‐γ, FABP4, Adipoq, and CD36—by qRT‐PCR (Figure S9A–D). Compared with the Control and US groups, Z‐H alone produced a moderate increase, whereas the Z‐H + US combination elicited a marked up‐regulation of all four genes. These comprehensive findings suggest that Z‐H@KGM‐XG Hydrogel, particularly when combined with US intervention, promotes skin wound healing through multiple mechanisms. Zinc ion release and microcurrent generation enhance cellular activities and positive regulation of cellular response to extracellular stimuli. The activation of the PPAR signaling pathway and associated metabolic regulation optimizes the wound‐healing microenvironment. The transcriptomic evidence provides a substantial biological basis for the therapeutic application of Z‐H@KGM‐XG hydrogel in wound management.

## 4 Conclusion

In conclusion, we constructed a nature‐based sonosensitizer for broad‐spectrum infectious tissue repair. We first prepared nanomaterials in different ratios and screened them for optimal composition. Subsequently, we investigated the therapeutic effects of microcurrents generated by low‐intensity US in synergy with Zn ions. We evaluated the repair capabilities in three distinct infectious microenvironments: infected bone defects, wound infections, and wound biofilm formation. To address the specific challenges of biofilm infections, the material was loaded into microneedles for deep, full‐thickness antibacterial treatment. Our research found that early‐stage high‐intensity US could rapidly eliminate all bacteria, while later stage low‐frequency US, through the synergy of microcurrents and Zn ions, promotes tissue repair. To further explore the repair mechanisms, we developed a biologically derived composite hydrogel loaded with nanoparticles with excellent biosafety and water retention properties. RNA sequencing revealed that the microcurrents in synergy with Zn ions promote repair through the PPAR signaling pathway. Therefore, this study highlights the potential of nature‐based sonosensitizers for improving sonocatalysis and opens new avenues in infected tissue repair through sono‐ionic therapy.

## Author Contributions

Liang Ma involved in Writing – original draft, visualization, and methodology. Hongchuan Wang and Weidong Liang were involved in Writing – review and editing, visualization, validation, and methodology. Tao Xu, Jie Lei, Qian Li, and Wenzhao Wang were involved in validation and methodology. Li Zhang, Shutao Gao, and Xiaoyu Cai were involved in methodology and investigation. Cao Yang and Yingsong Wang were involved in Writing – review and editing, supervision, and funding acquisition. Chuanhui Xun and Weibin Sheng were involved in validation, supervision, and resources.

## Ethics Statement

All animal experimental procedures were reviewed and approved by the Animal Research Committee of Tongji Medical College, Huazhong University of Science and Technology, Wuhan, China (Approval No. S2806).

## Conflicts of Interest

The authors declare no conflicts of interest.

## Supporting information




**Supporting File**: exp270208‐sup‐0001‐SuppMat.docx.

## Data Availability

The data that support the findings of this study are available from the corresponding author upon reasonable request.
