# Supplementary material for: Engineered Nature‐Based Sonosensitizer for Broad‐Spectrum Infectious Tissue Repair via Sono‐Ionic Therapy
Source: Exploration (Beijing). 2026 Aug 10;6(4):70208. doi: 10.1002/exp2.70208 (PMC13457404; doi:10.1002/exp2.70208)
Supplement: Supplementary file 1 [file EXP2-6-70208-s001.docx]

**Engineered** **nature-based** **Sonosensitizer for** **Broad-Spectrum Infectious Tissue Repair via Sono-Ionic Therapy**

Liang Ma^1^^*^, Hongchuan Wang^1*^, Weidong Liang^2*^, Tao Xu^2^, Jie Lei^1^, Li Zhang^3^, Shutao Gao^2^, Xiaoyu Cai^2^, Qian Li^4^, Wenzhao Wang^5^, Chuanhui Xun^2^, Weibin Sheng^2#^, Cao Yang^1#^, Yingsong Wang^3#^

1. Department of Orthopaedics, Union Hospital, Tongji Medical College, Huazhong University of Science and Technology, Wuhan, 430022, China;

2. Department of Spine Surgery, The First Affiliated Hospital of Xinjiang Medical University, Urumqi, 830054, Xinjiang, China;

3. Department of Orthopedics, The Second Affiliated Hospital of Kunming Medical University, 374# Dianmian Road, Kunming, Yunnan 650101, China;

4. Department of Chemical & Biomolecular Engineering, University of Notre Dame, Notre Dame, Indiana 46556 United States;

5. School of Biomedical Sciences, The Chinese University of Hong Kong, Hong Kong SAR, 999077, China.

^*^These authors contributed equally to this work

**^#^Correspondence:**

Weibin Sheng: wbsheng@vip.sina.com

Cao Yang：caoyangunion@hust.edu.cn

Yingsong Wang: ynwys@163.com

**Figures**

**Figure S1.** (A) TEM images and elemental mapping of Z-1H, Z-2H and Z-6H. Scar bar: 200 nm. Size distribution histograms of (B) ZIF-8 and (C) Z-6H nanoparticles calculated from SEM images. (D, E) *In vitro* antibacterial activity of different components under ultrasound irradiation. (D) Representative images of bacterial colonies after treatment with PBS (Ctrl), ZIF-8, Hb, and Z-6H, with or without ultrasound (US). (E) Quantitative analysis of bacterial survival by colony counting (CFU mL^-1^, log scale). Data are presented as mean ± SD (n = 3); ****p < 0.0001; ns = not significant.

**Figure S2.** Time-dependent Zn^2+^ release profiles of Z-H with (Z-H/US) or without US under identical pH and temperature.

**Figure S3. Sonocatalytic detection and ROS generation ability.** (A-C) The ^1^O_2_ generation of Z-1H, Z-2H and Z-6H under US detecting by the decrease of DMA fluorescence intensity. (D-F) Adsorption of NBT treated by Z-1H, Z-2H and Z-6H for 18 min under US. (G) The ^1^O_2_ generation of US alone detecting by the decrease of DMA fluorescence intensity. (H) Adsorption of NBT treated by US alone for 18 min.

**Figure S4.** HE staining of heart, liver, spleen, lung and kidney. n = 3 independent experiments per group. Scale bar = 100 μm.

**Figure S5. Treatment of osteomyelitis.** (A) In vitro anti-biofilm activity of microneedle (MN) evaluated by crystal violet staining. Biofilms were treated with PBS (Ctrl), blank MN, or Z-H–loaded microneedles (Z-H MN) in the absence (−US) or presence (+US) of ultrasound. (B) Immunofluorescence staining of inos and IL6 of wound tissue on Day14 (Scale bar = 50 µm) (C) Immunofluorescence staining of CD31 and α-SAM of wound tissue on Day14 (Scale bar = 50 µm) (D, E) Immunohistochemical staining of IL-6 and TNF-α. n = 3 independent experiments per group.

**Figure S6.** (A) Wound healing quantitative analysis of wounds over time. (B) Swelling ratio of the hydrogels over 80 h in PBS (pH 7.4, 37 °C). (C) Representative stress–strain curves from uniaxial compression tests. (D) Cyclic compression tests (10 cycles at 40% strain). n = 3 independent experiments per group, ****p < 0.0001.

**Figure S7.** (A) Expression of differential genes. (B) Volcano plot of GU vs G. n = 3 independent experiments per group.

**Figure S8.** (A) GO enrichment analysis of G vs C. (B) KEGG enrichment analysis of G vs C. n = 3 independent experiments per group.

**Figure S9.** (A, B, C, D) PPAR-γ, FABP4, Adipoq, and CD36 qRT-PCR results of hBMSCs treated in different conditions (Control, US, Z-H and Z-H +US) after 14 days. n = 3 independent experiments per group, *P< 0.05, **P < 0.01, ****P < 0.0001, ns = not significant.
